# Hybrid similarity relation based mutual information for feature selection in intuitionistic fuzzy rough framework and its applications

**DOI:** 10.1038/s41598-024-55902-z

**Published:** 2024-03-12

**Authors:** Anoop Kumar Tiwari, Rajat Saini, Abhigyan Nath, Phool Singh, Mohd Asif Shah

**Affiliations:** 1 Department of Computer Science and Information Technology, Central University of Haryana, Mahendergarh, 123031 India; 2 Department of Mathematics, School of Basic Sciences, Central University of Haryana, Mahendergarh, 123031 India; 3 Department of Biochemistry, Pt. Jawahar Lal Nehru Memorial Medical College, Raipur, 492001 India; 4 Department of Mathematics (SoET), Central University of Haryana, Mahendergarh, 123031 India; 5 Department of Economics, Kebri Dehar University, 250, Kebri Dehar, Somali, Ethiopia; 6 Centre of Research Impact and Outcome, Chitkara University Institute of Engineering and Technology, Chitkara University, Rajpura, 140401 Punjab India; 7 Division of Research and Development, Lovely Professional University, Phagwara, 144001 Punjab India

**Keywords:** Rough set, Granular structure, Intuitionisitic fuzzy relation, Intuitionistic Fuzzy Set, Mutual information, Computational biology and bioinformatics, Health care, Molecular medicine

## Abstract

Fuzzy rough entropy established in the notion of fuzzy rough set theory, which has been effectively and efficiently applied for feature selection to handle the uncertainty in real-valued datasets. Further, Fuzzy rough mutual information has been presented by integrating information entropy with fuzzy rough set to measure the importance of features. However, none of the methods till date can handle noise, uncertainty and vagueness simultaneously due to both judgement and identification, which lead to degrade the overall performances of the learning algorithms with the increment in the number of mixed valued conditional features. In the current study, these issues are tackled by presenting a novel intuitionistic fuzzy (IF) assisted mutual information concept along with IF granular structure. Initially, a hybrid IF similarity relation is introduced. Based on this relation, an IF granular structure is introduced. Then, IF rough conditional and joint entropies are established. Further, mutual information based on these concepts are discussed. Next, mathematical theorems are proved to demonstrate the validity of the given notions. Thereafter, significance of the features subset is computed by using this mutual information, and corresponding feature selection is suggested to delete the irrelevant and redundant features. The current approach effectively handles noise and subsequent uncertainty in both nominal and mixed data (including both nominal and category variables). Moreover, comprehensive experimental performances are evaluated on real-valued benchmark datasets to demonstrate the practical validation and effectiveness of the addressed technique. Finally, an application of the proposed method is exhibited to improve the prediction of phospholipidosis positive molecules. RF(h2o) produces the most effective results till date based on our proposed methodology with sensitivity, accuracy, specificity, MCC, and AUC of 86.7%, 90.1%, 93.0% , 0.808, and 0.922 respectively.

## Introduction

The current trend of accumulation of huge amount of data in different databases pertaining to different domains has given rise to the unique opportunity of knowledge discovery/extraction using a plethora of data mining techniques^1^. These techniques^2^ can be explored in three ways namely knowledge types, architecture types, and analysis types along with their powerful applications in distinct research and practical domains to solve the interesting real-world problems. Data Mining plays a vital role in establishing smart agriculture application tools to accomplish real-time data analysis with large volume of data. Data mining tasks^3^ offer essential hidden patterns, correlation, and knowledge from the various applications of bioinformatics datasets, viscous dissipation, and activation energy^4,5^. Machine learning methods provide a set of techniques that can be used to create prediction/discriminatory models and subsequent knowledge extraction, which may facilitate in decision making or for better understanding of the concerned domain^6,7^. The “curse of dimensionality” plagues the effectiveness of various machine learning algorithms, but the development of dimensionality reduction methods^8^ have considerably impacted in reducing the effects of redundancy present in high dimensional datasets. In the fields of data mining, signal processing, biomedical imaging, agriculture, industrial engineering, and bioinformatics, researchers frequently face obstacles due to “curse of dimensionality” as it leads to enlarge the cost of data storage and extensive computing^9^. Moreover, this issue directly affects both the efficiency and accuracy to cope with different problems^10^. Dimensionality reduction process can easily eliminate redundancy and/or irrelevancy, noise, minimize the complexity of machine learning methods, and enhance the overall accuracy of classification process, and can be identified as an essential and key phase in pattern recognition scheme^11^.

Redundant features affects negatively to the various machine learning algorithms mostly resulting in high computation time and less accurate predictive models^12^. It also complicates the model interpretation. Feature reduction methods can be used to mitigate the negative effects of high dimensional data by facilitating the selection of low dimensional non-redundant subset of features. Feature reduction methods have been found to be very effective in a wide variety of research areas, including biological domain^13,14^.

Most popular methods of feature reduction algorithms fall under filter and wrapper methods. While wrapper methods are classifier dependent for the evaluation of features^15,16^, filter methods use classifier independent feature selection criterion and are generally less computationally intensive^17^.

Previously rough set theory^18,19^ have been applied very promisingly in feature selection^20^. Although classical rough set theory based feature selection methods^21,22^ can only be used on discrete features, which makes it mandatory for discretization of continuous features^23,24^. There is a fair chance of information loss during the process of discretization^25^.

The combination of fuzzy^26^ and rough sets^27^ effectively deals with uncertainty, vague and incomplete data. Rough set theory has been competently employed to produce the most informative features from a dataset consisted of discretized conditional attribute values. This informative feature subset is produced from the original features set with minimum information loss, and termed as reduct. Rough set deals with vagueness, whilst fuzzy set handles uncertainty. Fuzzy set theory ensures that real-valued datasets can be handled without any further discretization. By combining fuzzy set with rough set, information loss due to discretization can be effectively avoided as fuzzy rough set (FRS) can handle real-valued information system (dataset) directly. FRS can be effectively used for mitigating the effects of information loss as a consequence of discretization of features by using fuzzy similarity measures to tackle the continuous feature values^28^. Broadly, FRS aided dimensionality reduction^29^ methods can be categorized into two types^30,31^ which are based on discernibility matrix and dependency function^32^. Discernibility matrix assisted approaches provide numerous reduct sets^33^, whilst dependency function leads to a single feature subset^34^.

In FRS aided dimensionality reduction theory, a similarity relation is incorporated between the data points to construct lower and upper approximations. By taking union of the computed lower approximations, we obtain the positive region of decision. Here, the wider is the obtained membership to positive region; greater is the plausibility of instance belonging to an individual category^35^. Based on dependency function, we compute significance of a subset of features. Moreover, the conditional entropy measure is employed in to calculate reduct set for both homogeneous and heterogeneous information system respectively^36–38^. However, it may lead to misclassification of samples when there is a large degree of imbricate between diverse categories of data. Also, it can cope with only with membership of data point to a set, where uncertainty cannot be handled due to both identification and justification. Hence, there is an essential and utmost requirement of distinct kind of mathematical model that can both fit data, and at the same moment it can tackle uncertainty emerging due to identification^39^.

Intuitionistic fuzzy (IF) set^40,41^ is step ahead that offers two degree of freedom by taking into consideration both membership and non-membership, which can cope with uncertainty that emerges both in judgement and identification^42^. It has been successfully exercised in decision making^43^, image segmentation, rule generation, and machine learning^44,45^. In the recent few years, the assemblage of IF^46^ and rough sets^47^ are employed to establish numerous IF rough set models^48,49^ to effectively handle later uncertainty and vagueness in the data^50,51^. Huang et al.^52^ proposed a ranking based model for selecting the neighbourhood of objects^53,54^ and presented a Dominant IF Decision Table (DIFDT)^55^ by using discernibility matrix and assisted discernibility function^23^. They developed IFRS based reduction technique for knowledge extraction from given information system. Huang et al.^56^ presented the IF multigranulation rough set (IFMGRS) model and studied different reduction techniques to eliminate redundant granules by introducing reducts for three different types of IFMGRSs in 2014. Tan et al.^57^ used the concept of granular structure to introduce an IF rough set model^58^ and employed it for feature selection. Tiwari et al.^59^ discussed an IF tolerance relation, which was applied to establish IF rough set aided feature selection. Shreevastava et al.^60^ addressed different similarity relation assisted technique to deal with both supervised and semi-supervised data. Tiwari et al.^61–63^ and Shreevastava et al.^64,65^ elaborated different issues related to feature selection technique and presented several lower and upper approximations by using various mathematical ideas. A feature selection to track multiple samples was presented by Li et al.^66^ by using IF clustering notion. IF quantifier was introduced by Singh et al.^67^ to construct IF rough set model and its application to feature reduction. Jain et al.^39^ tried to minimize noise in the data by using the concept of IF granules and incorporated different types of IF relations to introduce feature selection both robust and non-robust. From the recent published articles, it is conspicuous that the use of IF set theory assisted notion for feature selection is still in its incipient stage. Uncertainty is measured in terms of entropy and has its origin in the telecommunications domain^68,69^. Mutual information (MI)^70^ aims to measure the relationship between feature and the target. Further, it can be stated that mutual information (MI)^71^ is an interesting quantity that evaluates the dependence between conditional features and has been repeatedly employed to solve an extensive diverse problems. Feature selection techniques can be converted into effective one by incorporating information entropy estimation notion for attribute extraction based on MI^72^ and the conventional feature selection approaches on the basis of class seperability. Broadly MI measures the amount of information that can be deduced from a random variable/vector about another random variable/vector^73,74^.

Max-relevance-minimum-redundancy method^75,76^ is based on the concept of MI and has been relevant in a number of previous studies. It deduces the target MI with minimum redundancy^10,77^ among the selected features. A number of MI based feature selection algorithms have been in practice in various domains^72,74^. Fuzzy rough entropy was effectively used to avoid the limitation of rough entropy to handle the real-valued feature data^78,79^, but fuzzy rough entropy leads to lessening monotonically with the rise of the dimensions of data, which can promptly reflect the roughness of information systems. This issue was resolved up to certain extent by presenting the extension of fuzzy rough based information entropy with conditional entropy, joint entropy, and mutual information. However, none of the works has handled the noise, vagueness, and uncertainty due to both identification and judgement simultaneously, which is frequently appearing in the current era of high-dimensional datasets due to advancement of internet based technologies. In the current study, a new IFRS based joint entropy, conditional entropy, and mutual information based on a new IF hybrid relation and IF granular structure to handle the different issues such as later uncertainty, vagueness, and imprecision available in the large volume of high dimensional datasets that may degrade the performances of learning algorithms. Firstly, a novel hybrid IF similarity relation is presented. Secondly, joint and conditional entropies are established in IF rough framework. Thirdly, IF rough mutual information is introduced. Then, lower and upper approximations are computed by using presented hybrid IF similarity relation. Thereafter, dependency function is computed by using the defined lower approximation. Next, significance of feature subset is computed by using IF rough mutual information. Further, a heuristic feature selection algorithm is discussed by using both significance and dependency function. IF rough mutual information are employed to measure the later uncertainty and the correlation between features and class. Next, this algorithm is applied on benchmark datasets, and the reduct is computed. The effectiveness of the proposed algorithm is further explained by measuring the performances of seven widely used learning techniques on reduced data produced by our method and four existing approaches. Finally, the proposed method is applied to enhance the overall prediction to discriminate the phsopholipidosis^80^ positive (PL+) and phsopholipidosis negative (PL-) molecules. Phospholipidosis is a condition when there is an abnormal buildup of phospholipids in various tissues due to the usage of cationic amphiphilic pharmaceuticals. Phsopholipidosis (PPL) is a reversible condition, and phospholipidosis levels revert to normal once the cationic amphiphilic medications are stopped^81^. Computational prediction of possible inducing characteristics utilizing structure-activity relationship (SAR) can enhance the traditional high throughput screening and drug development pipelines because to its rapidity and cost-effectiveness^82^.The main contributions of the entire study can be highlighted as follows:

**Major contributions of the study**

- This study establishes a new hybrid IF similarity relation that can deal with both nominal and numerical features.
- An IF granular structure is presented to handle the noise in mixed data.
- IF rough entropy, joint entropy, and conditional entropy is given to handle the later uncertainty with information entropy.
- Further, the idea of an If rough mutual information is discussed.
- Moreover, this If rough mutual information is employed to evaluate both uncertainty and the correlation between conditional feature and decision class.
- Then, a feature selection approach is introduced by using this IF rough mutual information concept.
- Finally, a framework is designed based on our proposed methods to enhance the prediction of phospholipidosis positive molecules.

## Theoretical background

In this segment, few essential basic notions about IF set, IF relation, IF information system, and mutual information is reviewed. These concepts can be explained/described as follows:

### Definition 2.1

**IF set** An IF set X in ${\mathbb {U}}$ is well defined collection of samples/objects having the form

1 $$\begin{aligned} X = \bigg \{<x,\mu _{X}(x),\nu _{X}(x)> \bigg |\forall x \in {\mathbb {U}} \bigg \} \end{aligned}$$

where, ${\mathbb {U}}$ portrays the set of data points/samples/objects. Moreover, $\mu _{X}:{\mathbb {U}}\rightarrow [0,1]$ along with $\nu _{X}:{\mathbb {U}}\rightarrow [0,1]$, which holds the essential condition $0\le \mu _{X}(x) + \nu _{X}(x)\le 1, \forall x \in {\mathbb {U}}$. Here, $\mu _{X}(x)$ and $\nu _{X}(x)$ are depicted as the imperative membership and non-membership grades for a given element $x \in {\mathbb {U}}$. Further, $\pi _{X}(x)= 1 - \mu _{X}(x) -\nu _{X}(x)$ portrays the hesitancy grade of $x \in {\mathbb {U}}$. Additionally, we have $0\le \pi _{X}(x) \le 1$, $\forall x \in$ ${\mathbb {U}}$. Thus, the obtained ordered pair $<\mu _{X},\nu _{X}>$ is depicted as a requisite IF value.

### Definition 2.2

**IF information system** An IF information system (IFIS) can be exemplified by a quadruple ( $${\mathbb {U}}$$,$$ C, V_{IF},IF)$$, where, we have $$V_{IF} $$, which is comprised of all IF values. Further, we have a mapping, which can be portrayed by *IF* :  $${\mathbb {U}}$$ $$\times C\rightarrow V_{IF}$$, in such a way that $$IF(x, a) = <\mu _{X}(x),\nu _{X}(x)>$$,$$\forall x \in {\mathbb {U}}$$, $$ \forall a\in C$$.

### Definition 2.3

**IF relation** Let $R(x_i,x_j)=(\mu _X(x_i,x_j),\nu _X(x_i,x_j))$ be an IF binary relation induced on the system. $R(x_i,x_j)$ is IF similarity relation if it satisfies :

1. Reflexivity: For any given *i* and *j*,
  2 $$\begin{aligned} \mu _{R}(x_i,x_j)=1~~{\text {and}}~~\nu _{R}(x_i,x_j)=0 \end{aligned}$$
2. Symmetry: For any given *i* and *j*,
  3 $$\begin{aligned} \mu _{R}(x_i,x_j)=\mu _{R}(x_j,x_i)~~{\text {and}}~~\nu _{R}(x_i,x_j)=\nu _{R}(x_j,x_i) \end{aligned}$$
  $$\forall x_i,x_j \in {\mathbb {U}}$$

### Definition 2.4

**Mutual information** Mutual information (MI) can be expresserd based on broadely depicted entropy and well-known conditional entropy by using the following given equation

4 $$\begin{aligned} I(P;D)=H(D)-H(D|P) \end{aligned}$$

where, $P \subseteq C$, H(D) and H(D|P) depict information entropy and conditional entropy respectively. Decrease of uncertainty about D gernerated by P is evaluated by mutual information and its inverse is computed in the same way. Mutual information is employed to calculate either volume of information of P enclosed in D or D included in P. H(P) is amount of information contained in P about itself which means I(P;P)=H(P)

### Definition 2.5

**Significance of conditional feature** For a given IFIS and $B \subseteq C$, if we have an arbitrary conditional dimension/feature $b\in (C-B)$, then its significance can be illustrated by the following equation

5 $$\begin{aligned} SGF(b,B,D)=I(B\cup {b};D)-I(B;D)=H(D|B)-H(D|B\cup {b}) \end{aligned}$$

and $$B=\phi $$, $$ SGF(b,B,D) =H(D)-H(D|{b})=I({b};D)$$, which is a MI between conditional dimension/feature *b* and decision feature *D*. If the calculated value of SGF(b, B, D) is greater, then it insinuates that under the known condition of feature subset *B*, dimension *b* is found to be more potential for the available decision feature *D*.

## Proposed work

In the underway segment, we demonstrate a hybrid IF similarity relation, granular structure, and MI. Based on these concepts, a feature selection procedure is introduced to discard irrelevancy and redundancy available in the high-dimensional information systems.

**IF Relation:** For all $a \in C$, and $x_i,x_j \in {\mathbb {U}}$, the hybrid similarity $R_{a}^h\left( x_i,x_j\right)$ between $x_i$ and $x_j$ with respect to any given *a* can be defined by:

6 $$\begin{aligned} R_{a}^h(x_i, x_j) ={\left\{ \begin{array}{ll} 1, &{} a(x_i) = a(x_j) \text { and } a \text { is nominal;} \\ 0, &{} a(x_i) \ne a(x_j) \text { and } a \text { is nominal;} \\ 1 -\frac{1}{n^2} \displaystyle \sum _{i=1}^{n} \sum _{j=1}^{n}(|\mu _{a}(x_{i}) - \mu _{a}(x_{j})|\\ \hspace{2.5cm}\times |\nu _{a}(x_{i}) - \nu _{a}(x_{j})| ), &{} a \text { is numerical and } |\mu _{a}(x_{i}) - \mu _{a}(x_{j})| \le \zeta _a\\ &{}|\nu _{a}(x_{i}) - \nu _{a}(x_{j})|>\zeta _a;\\ 0, &{} a \text { is numerical and } |\mu _{a}(x_{i}) - \mu _{a}(x_{j})| > \zeta _a\\ &{}|\nu _{a}(x_{i}) - \nu _{a}(x_{j})| \le \zeta _a;\\ \end{array}\right. } \end{aligned}$$

where, $\zeta _a=1-R_{a}^h(x_i, x_j)$ is depicted as an adaptive IF radius. The IF relation and IF relation matrix enticed by *a* $\in$ ${\mathbb {U}}$ are $R_{a}^h$ and $M_{R_{a}^h} = \left( r_{ij}\right) _{n \times n}$, where $r_{ij} = R_{a}^h\left( x_i, x_j\right)$.

If we have $C_1 = \{a_1,a_2,\dots ,a_{|C_1} \}\subseteq {\mathbb {C}}$, then,

7 $$\begin{aligned} R_{C_1}^h\left( x_i, x_j\right) = \bigwedge \limits _{l=1}^{|C_1|} R_{a}^h\left( x_i, x_j\right) \end{aligned}$$

### Proof

1. Reflexive: If we take a case when $x_i=x_j$, then, proposed relation follows only two cases, which are first and third. Moreover, other two cases are rejected by default.
  Case 1. if $a(x_i) = a(x_j)$ where *a* is a nominal , then we obtain $R_{a}^h(x_i,x_j)$=$R_{a}^h(x_i,x_i)$=1
  Case 2. If *a* is numerical and $|\mu _{a}(x_{i}) - \mu _{a}(x_{j})| \le \zeta _a$ and $|\nu _{a}(x_{i}) - \nu _{a}(x_{j})| >\zeta _a$,then $R^{h}_a\left( x_i,x_j\right) =1 -\frac{1}{n^2} \displaystyle \sum _{j=1}^{n} \sum _{i=1}^{n}(|\mu _{a}(x_{j}) - \mu _{a}(x_{i})||\nu _{a}(x_{j}) - \nu _{a}(x_{i})|)$
  Now,if we put $x_i=x_j$, we get the folllowing results:
$$R^{h}_a(x_i,x_i)=1 -\frac{1}{n^2} \displaystyle \sum _{i=1}^{n}(|\mu _{a}(x_{i}) - \mu _{a}(x_{i})||\nu _{a}(x_{i}) - \nu _{a}(x_{i})|)$$
  $R^{h}_a(x_i,x_i)=1$, therefore, we get $R^{h}_a\left( x_i,x_j\right)$ as refelxive
2. Symmetry:
  8 $$\begin{aligned}&R_{a}^h\left( x_i, x_j\right) ={\left\{ \begin{array}{ll} 1, &{} a(x_i) = a(x_j) \text { and } a \text { is nominal;} \\ 0, &{} a(x_i) \ne a(x_j) \text { and } a \text { is nominal;} \\ 1 -\frac{1}{n^2} \displaystyle \sum _{i=1}^{n} \sum _{j=1}^{n}(|\mu _{a}(x_{i}) - \mu _{a}(x_{j})|\\ \hspace{2.5cm}\times |\nu _{a}(x_{i}) - \nu _{a}(x_{j})| ), &{} a \text { is numerical and } |\mu _{a}(x_{i}) - \mu _{a}(x_{j})| \le \zeta _a\\ &{}|\nu _{a}(x_{i}) - \nu _{a}(x_{j})|>\zeta _a;\\ 0, &{} a \text { is numerical and } |\mu _{a}(x_{i}) - \mu _{a}(x_{j})| > \zeta _a\\ &{}|\nu _{a}(x_{i}) - \nu _{a}(x_{j})| \le \zeta _a;\\ \end{array}\right. } \end{aligned}$$
  9 $$\begin{aligned}&\quad R_{a}^h\left( x_i, x_j\right) ={\left\{ \begin{array}{ll} 1, &{} a(x_j) = a(x_i) \text { and } a \text { is nominal;} \\ 0, &{} a(x_j) \ne a(x_i) \text { and } a \text { is nominal;} \\ 1 -\frac{1}{n^2} \displaystyle \sum _{j=1}^{n} \sum _{i=1}^{n}(|\mu _{a}(x_{j}) - \mu _{a}(x_{i})|\\ \hspace{2.5cm}\times |\nu _{a}(x_{j}) - \nu _{a}(x_{i})|), &{} a \text { is numerical and } |\mu _{a}(x_{j}) - \mu _{a}(x_{i})| \le \zeta _a\\ &{}|\nu _{a}(x_{j}) - \nu _{a}(x_{i})|>\zeta _a;\\ 0, &{} a \text { is numerical and } |\mu _{a}(x_{j}) - \mu _{a}(x_{i})| > \zeta _a\\ &{}|\nu _{a}(x_{j}) - \nu _{a}(x_{i})| \le \zeta _a;\\ \end{array}\right. } \end{aligned}$$
  Now, it can be identified that
  $$\begin{aligned} R_{a}^h\left( x_i, x_j\right) =R_{a}^h\left( x_j, x_i\right) \end{aligned}$$
  So , $R_{a}^h\left( x_i, x_j\right)$ is symmetric

Since, $R_{a}^h\left( x_i, x_j\right)$ is both reflexive and symmetric. Hence, we can obviously conclude that $R_{a}^h\left( x_i, x_j\right)$ is an IF similarity relation. $\square$

### Granular structure

The IF granule $\forall x_i\in {\mathbb {U}}$ is elicited by $C_1$ as follows:

10 $$\begin{aligned} \mu _{{[X_i]}_p^\varepsilon } (x_j)= {\left\{ \begin{array}{ll} 0,&{} \mu _{{R_p}^h}(x_i,x_j)<\epsilon \\ \,\,\,\, \,\,&{}\hspace{4cm}x_j \in {\mathbb {U}}\\ \,\,\mu _{{R_p}^h}(x_i,x_j),&{} \mu _{{R_p}^h}(x_i,x_j)\ge \epsilon \end{array}\right. } \end{aligned}$$,

further,

11 $$\begin{aligned} \nu _{{[X_i]}_p^\epsilon } (x_j)= {\left\{ \begin{array}{ll} 0,&{} \nu _{{R_p}^h}(x_i,x_j)<\epsilon \\ \,\,\,\, &{}\hspace{4cm}x_j \in {\mathbb {U}}\\ \,\,\nu _{{R_p}^h}(x_i,x_j),&{} \nu _{{R_p}^h}(x_i,x_j)\ge \epsilon \end{array}\right. } \end{aligned}$$

$\forall a \in P$ is subset of C and $\epsilon \in [0,1]$

By using IF granulation structure, rough entropy can be discussed into IF rough framework, and IF rough entropy of a feature can be described by:

#### Definition 3.1

The IF rough entropy of $C_1$ can be given as:

12 $$\begin{aligned} ET(C_1)= ET\left( R_{C_1}^h\right) =-\frac{1}{n} \sum _{i=1}^n \log _2 \frac{1}{\left| [x_i]_{R_{C_1}^h}\right| } \end{aligned}$$

It is obvious to identify that $0 \le ET(C_1)\le \log _2n$ iff $\forall x_i,x_j \in {\mathbb {U}}, R_{C_1}^h(x_i,x_j)=1, \left| [x_i]_{R_{C_1}^h}\right| =n ,$ so $ET(C_1)=\log _2 n$. In this suit all the sample pairs are found to be identical. Therefore, the obtained granulation space is found to be the largest at this time, on the contrary $\forall x_i\ne x_j \hspace{0.1cm} R_{C_1}^h(x_i,x_j)= 0,$ which indicates $\left| [x_i]_{R_{C_1}^h}\right| =1$. Therefore, $ET(C_1)=\log _2 n = 0$. Now,the granulation space is instituated as the smallest one.

#### Definition 3.2

The IF joint rough entropy of $C_1$ and $C_2$ can be expressed by :

13 $$\begin{aligned} ET(C_1,C_2) = ET\left( R_{{C_1\cup C_2}}^h \right) = -\frac{1}{n} \sum _{i=1}^n \log _2 \frac{1}{\left| [x_i]_{R_{C_1}^h} \cap [x_i]_{R_{C_2}^h}\right| }. \end{aligned}$$

#### Definition 3.3

The IF rough conditional entropy of $C_2$ relative to $C_1$ can be addressed by the following equation :

14 $$\begin{aligned} ET(C_2 |C_1) = -\frac{1}{n} \sum _{i=1}^n \frac{\left| [x_i]_{R_{C_1}^h}\right| }{\left| [x_i]_{R_{C_1}^h}\right| \cap \left| [x_i]_{R_{C_2}^h}\right| } \end{aligned}$$

#### Definition 3.4

The IF rough mutual information of $$C_2 $$ and $$C_1$$ can be computed as follows;

15 $$\begin{aligned} I(C_2;C_1) = - \frac{1}{n} \sum _{i}^n \log _2 \frac{\left| [x_i]_{R_{C_1}^h}\right| \cap \left| [x_i]_{R_{C_2}^h}\right| }{\left| [x_i]_{R_{C_1}^h}\right| \left| [x_i]_{R_{C_2}^h}\right| } \end{aligned}$$

#### Definition 3.5

The IF rough mutual information between D and $C_1$ can be illustrated by the equation:

16 $$\begin{aligned} I(D;C_1)= - \frac{1}{n} \sum _{i=1}^n \log _2 \frac{\left| [x_i]_{R_{C_1}^h} \cap [x_i]_D\right| }{\left| [x_i]_{R_{C_1}^h}\right| |[x_i]_D|} \end{aligned}$$

By using this equation, IF rough mutual information $$ I(d;C_1)$$ considers as the correlation between $$C_1$$ and decision feature D . If the obtained value of IF rough mutual information between *D* and $$C_2$$ is higher, then, we get more correlated value between $$C_1$$ and *D*.

#### Proposition 3.6

If $$C_1\subseteq C_2 \subseteq C$$, then $$R_{C_1}^h \supseteq R_{C_2}^h$$

#### Proof

As discussed by the aforesaid definition 3.1, $R_{c_1}^h(x_i,x_j)=\bigwedge \limits _{l=1}^{|C_1|}R_{C_1}^h (x_i,x_j)$, $R_{c_2}^h(x_i,x_j)=\bigwedge \limits _{l=1}^{|C_2|}R_{C_2}^h (x_i,x_j)$ and $|C_1|\le |C_2|$ $\Rightarrow R_{c_2}^h(x_i,x_j)\subseteq R_{C_1}^h (x_i,x_j)$ $\Rightarrow$ $R_{C_1}^h \supseteq R_{C_2}^h$

Now, $$R_{C_1}^h \supseteq R_{C_2}^h \Longleftrightarrow \forall x_i,x_j \in {\mathbb {U}}$$;

$$R_{C_1}^h (x_i,x_j)\ge R_{C_2}^h(x_i,x_j)$$ $$\square $$

#### Proposition 3.7

If $$R_{C_1}^h \subseteq R_{C_2}^h $$, then $$ET\left( R_{C_1}^h \right) \le ET\left( R_{C_2}^h\right) $$.

#### Proof

For a given $R_{C_1}^h \subseteq R_{C_2}^h$, we have $\forall x_i,x_j\in {\mathbb {U}}$. Now, we can simply write $R_{C_1}^h(x_i,x _j) \le R_{c_2}^h(x_i,x_j)$ $\Rightarrow \left| [x_i]_{R_{C_1}^h} \right| \le \left| [x_i]_{R_{C_2}^h} \right|$

Therefore, we detect the result by using the definition 3.1 as $ET\left( R_{C_1}^h \right) \le ET\left( R_{C_2}^h\right)$. $\square$

#### Proposition 3.8

IF $$C_1\subseteq C_2 \subseteq C$$ then $$ ET(C_1) \ge ET(C_2)$$

#### Proof

For any given $C_1 \subseteq C_2$, we have the following expression based on the Proposition 3.6,

$R_{C_1}^h \supseteq R_{c_2}^h$. Moreover, by using Proposition 3.7, we can conclude the following result:

$$ET(C_1) \ge ET(C_2)$$

Proposition 3.8 depcits that IF rough entropy reduces when feature subset accquire larger size, whilst,it grows in case of features subset procures smaller size . It can be easily observed that IF rough entropy definition can evaluate the uncertainty of IF approximation space. $\square$

#### Proposition 3.9

Suppose $$C_1,C_2\subseteq C $$, then $$ET(C_1,C_2)\le \text {min} [ET(C_1),ET(C_2)]$$

#### Proof

Since $\forall x_i \in {\mathbb {U}}$ $[x_i]_{R_{C_1}^h} \cap [x_i]_{R_{C_2}^h} \subseteq [x_i]_{R_{C_1}^h}$ and $[x_i]_{R_{C_1}^h} \cap [x_i]_{R_{C_2}^h} \subseteq [x_i]_{R_{C_2}^h}$ $\Rightarrow \left| [x_i]_{R_{C_1}^h} \cap [x_i]_{R_{C_2}^h} \right| \le \left| [x_i]_{R_{C_1}^h} \right|$ and $\left| [x_i]_{R_{C_1}^h} \cap [x_i]_{R_{C_2}^h}\right| \le \left| [x_i]_{R_{C_2}^h}\right| .$ By Proposition 3.2, we have $ET(C_1,C_2) \le ET(C_1)$ and $ET(C_1,C_2)\le ET(C_2) .$ $\Rightarrow ET(C_1,C_2) \le \text {min} (ET(C_1),ET(C_2)).$ $\square$

#### Proposition 3.10

IF $$C_1\subseteq C_2 \subseteq C$$, then $$ ET(C_1,C_2) = ET(C_2)$$

#### Proof

Since $C_1 \subseteq C_2$, hence, by using the Proposition 3.6, we get

$$ R_{C_1}^h\supseteq R_{C_2}^h \Rightarrow [x]_{R_{C_1}^h}\supseteq [x]_{R_{C_2}^h} \Rightarrow [x]_{R_{C_1}^h} \cap [x]_{R_{C_2}^h}=[x]_{R_{C_2}^h} $$ So, $$ET(C_1,C_2)=ET(C_2) $$ $$\square $$

According to the Proposition 3.10, when there are two IF granules produced by two potential feature subsets respectively, then IF joint rough entropy of the calculated two potential feature subsets is equal to the IF rough entropy of the feature subsets corresponding to relatively smaller IF granulation.

#### Proposition 3.11

$$ ET(C_2 |C_1)= ET(C_2,C_1)- ET(C_1)$$.

#### Proof

Based on the Definition 3.3, we have $ET(C_1)+ ET(C_2 |C_1)= -\frac{1}{n} \sum _{i=1}^n \log _2 \frac{1}{|[x_i]_{R_{C_1}^h |}}-\frac{1}{n} \sum _{i=1}^n \frac{|[x_i]_{R_{C_1}^h}|}{|[x_i]_{R_{C_1}^h}| \cap |[x_i]_{R_{C_2}^h}|}$ $\Rightarrow ET(C_1)+ ET(C_2|C_1)=-\frac{1}{n}\left[ \sum _{i=1}^n\left[ \log _2 \frac{1}{|[x_i]_{R_{C_1}^h |}} + \log _2 \frac{|[x_i]_{R_{C_1}^h}|}{|[x_i]_{R_{C_1}^h}| \cap |[x_i]_{R_{C_2}^h}|}\right] \right]$ $\Rightarrow ET(C_1)+ET(C_2 |C_1)=- \frac{1}{n} \sum _{i=1}^n\left[ \log _2\frac{|[x_i]_{R_{C_1}^h |}}{|[x_i]_{R_{C_1}^h |}| |[x_i]_{R_{C_1}^h}| \cap |[x_i]_{R_{C_2}^h}| |}\right]$ $\Rightarrow ET(C_1)+ ET(C_2 |C_1)=- \frac{1}{n} \sum _{i=1}^n \log _2 \frac{1}{|[x_i]_{R_{C_1}^h} \cap [x_i]_{R_{C_2}^h}|}$ $\Rightarrow ET(C_1) + ET(C_2|C_1) = E(C_1,C_2)$ $\Rightarrow ET(C_2|C_1) = ET(C_1,C_2) - ET(C_1)$ $\square$

#### Proposition 3.12

If $$C_1\subseteq C_2 \subseteq C$$, then $$ ET(C_2|C_1)=0 $$

#### Proof

Since, $C_2 \subseteq C_1$ , hence, based on the Proposition 3.6, we can conclude that$R_{C_1}^h \subseteq R_{C_2}^h$. Therefore, $\forall x_i$, $[x_i]_{R_{C_1}^h} \subseteq [x_i]_{R_{C_2}^h}$, furthermore, $\forall x_{i}$ , $x_i,[x_i]_{R_{C_1}^h} \cap [x_i]_{R_{C_2}^h} = [x_i]_{R_{C_1}^h}$, now, based on the Definition 3.3, we have $ET(C_2|C_1) =-\frac{1}{n} \sum _{n=1}^n \log _2 \frac{\left| [x_i]_{R_{C_1}^h}\right| }{\left| [x_i]_{R_{C_1}^h}\right| \cap \left| [x_i]_{R_{C_2}^h}\right| }$ $\Rightarrow ET(C_2|C_1) =-\frac{1}{n} \sum _{n=1}^n \log _2 \frac{\left| [x_i]_{R_{C_1}^h}\right| }{\left| [x_i]_{R_{C_1}^h}\right| } = -\frac{1}{n} \sum _{n=1}^n \log _2 1 =0$ $\square$

IF rough mutual information can’t only be used to measure the uncertainty of IF approximation space but also can be applied to evaluate the correlation between conditional feature and decision class.

#### Proposition 3.13

$$I(C_1; C_2)= ET(C_2)-ET(C_2|C_1)$$ $$ = ET(C_1)-ET(C_1|C_2)$$

#### Proof

Based on the Proposition 3.9, we have $ET(C_2)-ET(C_2|C_1) =-\frac{1}{n} \sum _{i=1}^n \log _2 \frac{1}{\left| [x_i]_{R_{C_2}^h}\right| } + \frac{1}{n} \sum _{i=1}^n \log _2 \frac{\left| [x_i]_{R_{C_1}^h}\right| }{\left| [x_i]_{R_{C_1}^h} \cap [x_i]_{R_{C_2}^h} \right| }$ $\Rightarrow ET(C_2)-ET(C_2|C_1) =- \frac{1}{n} \sum _{i=1}^n \left[ \log _2 \frac{1}{\left| [x_i]_{R_{C_2}^h}\right| } - \log _2 \frac{\left| [x_i]_{R_{C_1}^h}\right| }{\left| [x_i]_{R_{C_1}^h} \cap [x_i]_{R_{C_2}^h} \right| } \right] \Rightarrow ET(C_2)-ET(C_2|C_1) = - \frac{1}{n} \sum _{i=1}^n \log _2 \frac{\left| [x_i]_{R_{C_1}^h} \cap [x_i]_{R_{C_2}^h} \right| }{\left| [x_i]_{R_{C_1}^h} \right| \left| [x_i]_{R_{C_2}^h} \right| } = I(C_1; C_2)$ Similarly, we can get $I(C_1; C_2)= ET(C_1)- ET(C_1 |C_2)$ $\square$

#### Proposition 3.14

$$ I(C_1; C_2)= I(C_2;C_1)= ET(C_1) +ET( C_2)-ET(C_1,C_2)$$

#### Proof

Obviously $I(C_1; C_2) = I(C_2; C_1)$ satisfies based on the Definitions 3.1, 3.4, and 3.5. Now, we obtain the following results: $ET(C_1) +ET( C_2)-E(C_1,C_2)=- \frac{1}{n} \sum _{i=1}^n \log _2 \frac{1}{\left| [x_i]_{R_{C_1}^h}\right| } -\frac{1}{n} \sum _{i=1}^n \log _2 \frac{1}{\left| [x_i]_{R_{C_2}^h}\right| } + \sum _{i=1}^n \log _2 \frac{1}{\left| [x_i]_{R_{C_1}^h} \cap [x_i]_{R_{C_2}^h} \right| }$ $\Rightarrow ET(C_1) +ET( C_2)-ET(C_1,C_2)= -\frac{1}{n} \sum _{i=1}^n \left[ \log _2 \frac{1}{\left| [x_i]_{R_{C_1}^h} \right| } + \log _2 \frac{1}{\left| [x_i]_{R_{C_2}^h} \right| } -\log _2 \frac{1}{\left| [x_i]_{R_{C_1}^h} \cap [x_i]_{R_{C_2}^h} \right| } \right]$ $\Rightarrow ET(C_1) +ET( C_2)-ET(C_1,C_2)=-\frac{1}{n} \sum _{i=1}^n \log _2 \frac{\left| [x_i]_{R_{C_1}^h} \cap [x_i]_{R_{C_2}^h} \right| }{\left| [x_i]_{R_{C_1}^h}\right| \left| [x_i]_{R_{C_2}^h} \right| } =I(C_1; C_2)$ $\square$

#### Definition 3.15

For a given IFIS, let P be subset of conditional dimensions/features(C).Thereafter,$\forall Y\in (C-P)$ is found to be the significance as $\Omega (Y,P,D)$, which can be further given by:

17 $$\begin{aligned} \Omega (Y,P,D)=I(P\cup {Y};D)-I(Y;D) \end{aligned}$$

$Y=\phi , \Omega (T,P,D)$, and can be outlined as, $\Omega (Y,D) =ET(D)- ET(D|{Y})=I({Y};D)$, which depicts the MI of IF conditional feature *T* and the decision feature *D*. If the value of $\Omega (T,P,D)$ increases, then IF conditional dimension/feature *T* is obtained to be more relevant for a given decision feature *D*.

**Algorithm 1:**
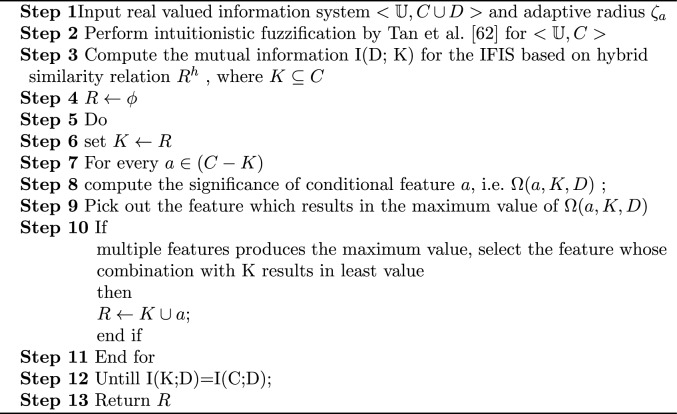
Feature selection alogrithm based on IF mutual information (FSIFMI)

## Experimentation

In the current experimental section, the performance of the proposed method is evaluated and compared with the existing fuzzy and IF sets assisted techniques. All the pre-processing concepts are implemented in Matlab 2023^83^ and learning algorithms are implemented in WEKA^84^. Firstly, fuzzification and intuitionistic fuzzification of the real valued data is performed by using the methods proposed by Jensen et al.^6^ and Tan et al.^57^ respectively. Secondly, the reduced datasets are obtained by the previously presented approaches. Thirdly, different threshold parameters values are adjusted for our established method to produce the reduct. Then, reduced datasets are generated by discarding the noise to the maximum level. The reduct is computed by changing the value of $\xi$ from 0.1 to 0.8 in small interval, and the value of $\xi$ providing the maximum performance measures in the experiment is selected as the final one. To perform the entire experimental study, the following setup is exercised to conduct the comprehensive experiments:

### Dataset

Ten benchmark datasets are taken from widely discussed University of California from Irvine based Machine Learning Repository^85^ to conduct the entire experiments. The required details of these datasets are outlined in Table 1. The dimension and size of these datasets depict that these are small to large datasets as number of data points range from 62 to 4521 and features range from 9 to 10000.

### Classifiers

Seven different learning methods^86^ are applied to demonstrate the performance measures on the reduced datasets obtained from different feature selection techniques. RealAdaBoost with random forest as base classifier (RARF) and IBK are employed for the objective of evaluating overall classification accuracies with standard deviation by using diverse validation techniques for ten benchmark reduced datasets. Moreover, we applied naive bayes, SMO, IBK, RARF, PART, JRip,J48, and random forest (RF) to evaluate the performances based on various evaluation metrics for the reduced Nath et al.^87^ dataset for evaluating the effectiveness of the proposed technique when compared to existing method for discriminating PL+ and PL- molecules.

**Dataset split**: Feature selection process is carried out over complete information system. After production of reduced datasets, individual learning algorithm is evaluated based on percentage split of 66:34 and kd-fold cross validation. In percentage split technique, dataset is randomly divided into two parts, where training is done on 66% of the entire dataset, while 34% of the dataset is employed to perform testing. In kd-fold cross validation, whole dataset is randomly separated into kd subsets, where kd-1 parts form training set, whilst one is employed to form testing set. After kd such repetitions, average value of different evaluation metrics is considered as final performance. In the current study, the value of kd is taken as 10.

### Performance evaluation metrics

The prediction performance measures of the seven learning algorithms from different categories are evaluated using both broadly elaborated threshold-dependent and threshold- independent assessment parameters. These assessment parameters are ascertained based on the calculated values of true positive (TRP), true negative (TRN), false positive (FLP), and false negative (FLN). TRP is computed number of correctly predicted positive data points; TRN is calculated number of correctly predicted negative data points. FLN is representation for the number of incorrectly predicted positive samples, while FLP is depiction for the number of incorrectly predicted negative samples. We employ different parameters namely: Sensitivity (Sn), Specificity (Sp), Accuracy (Ac), AUC, and MCC to measure the overall performances of the individual learning algorithms. Now, these evaluation parameters can be mathematically discussed as follows:

Sn: This calculates the overall percentage of correctly classified PPL+, which is specified by:

18 $$\begin{aligned} Sn=\frac{TRP}{(TRP+FLN)}\times 100 \end{aligned}$$

Sp: This includes the efficacious percentage of correctly classified PPL−, which is produced by:

19 $$\begin{aligned} Sp=\frac{TRN}{(TRN+FLP)}\times 100 \end{aligned}$$

Ac: The percentage of required overall correctly classified PPL+ and PPL− , which can be stated as:

20 $$\begin{aligned} Ac=\frac{TRP+TRN}{(TRP+FLN+TRN+FLP)}\times 100 \end{aligned}$$

AUC: It is applied to observe the important and required area under the receiver operating characteristic curve (ROC), the more tends its count towards 1, the better will be the obtained predictor.

MCC: Mathew’s correlation coefficient is a very much potential and the most awaited parameters, which is computed with the help of following equation:

21 $$\begin{aligned} MCC=\frac{TRP\times TRN-FLN\times FLP}{\sqrt{((TRP+FLP)(TRP+FLN)(TRN+FLN)(TRN+FLP)}}\times 100 \end{aligned}$$

This parameter is applied not only to clarify the effectiveness of the binary classifications but also to justify its efficiency. An MCC value tends towards 1 to specify that the predictor is the promising one.

### Results and discussion

**Figure 1:**
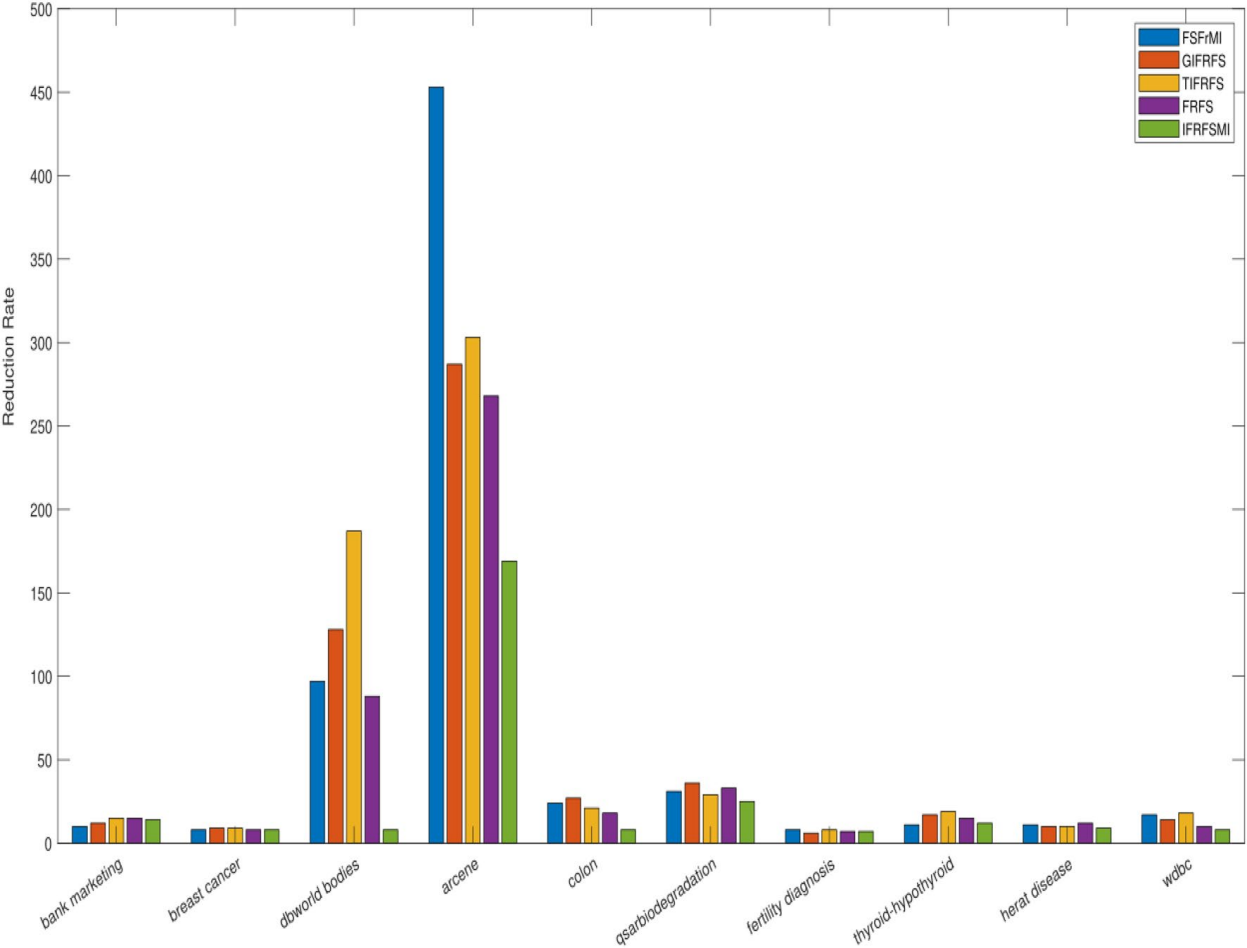
Comparison of overall reduction for different daasets by previous and proposed methods.

**Figure 2:**
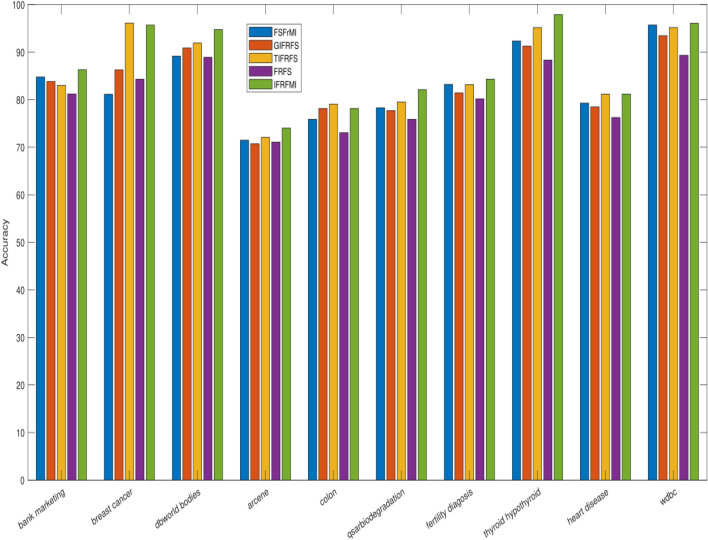
Comparison of average accuracies by IBK for different reduced datasets as produced by existing and proposed methods.

**Figure 3:**
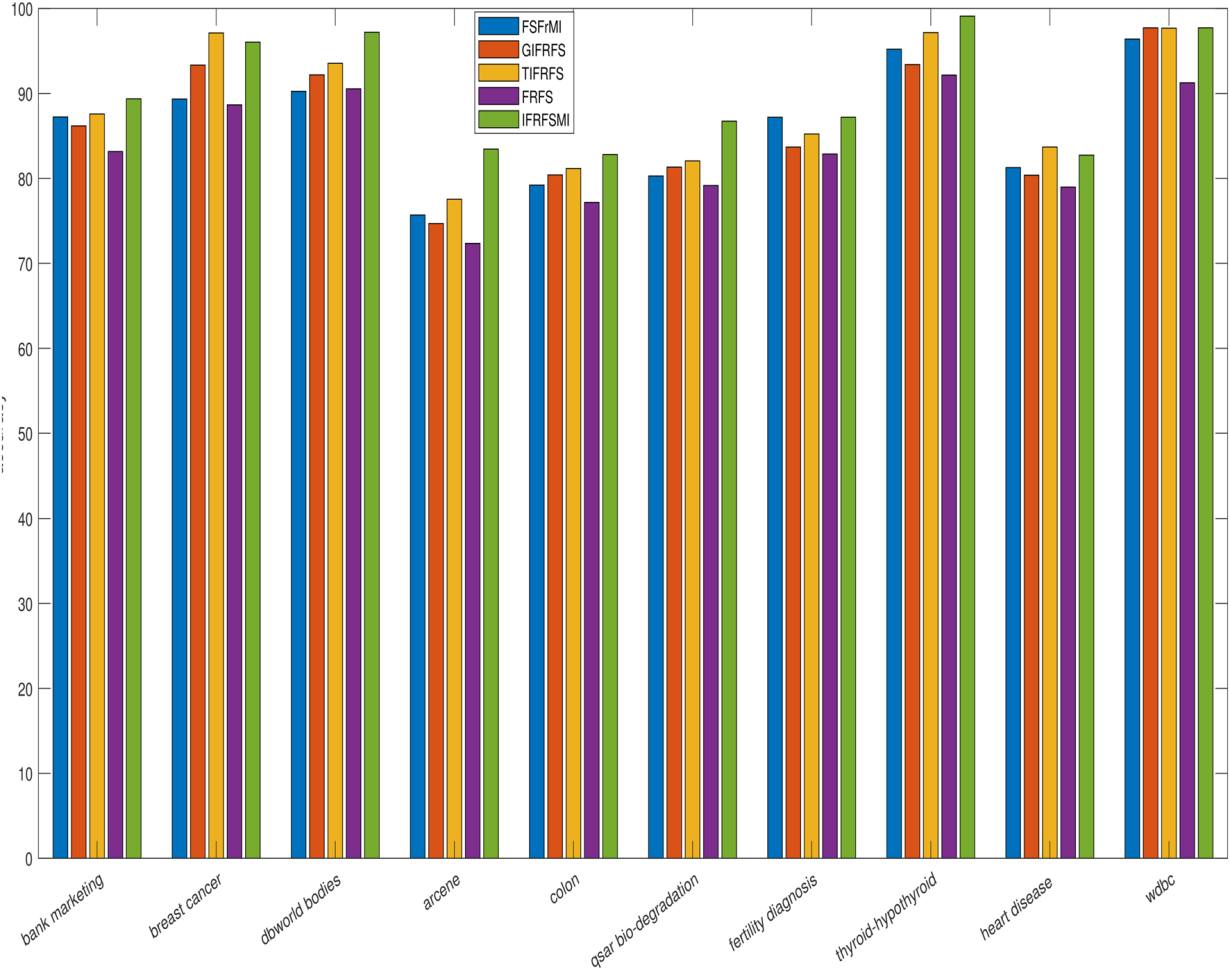
Comparison of average accuracies by RARF for different reduced datasets as produced by existing and proposed methods.

The details of the ten benchmark datasets along with the reduct as produced by four existing as well as presented methods is depicted in Table 1. Real-valued datasets are converted into fuzzy and IF values by using widely discussed Jensen et al.^6^ and Tan et al.^57^ concepts. Entire reduction process is accomplished over complete data by using both fuzzy and IF aided techniques. FSFrMI^72^, GIFRFS^57^, TIFRFS^59^, and FRFS^6^ are the earlier efficacious and effective techniques, which are incorporated to perform the comparative results (Table 2). Our proposed method produced reduct set range from 7 to 169, where reduct size is smaller when compared to reduct size by earlier approaches, except bank marketing and thyroid-hypothyroid datasets. For bank marketing dataset, FSFrM and GIFRFS resulted in relatively less size data, whilst smaller size is produced by FSFrMI and FRFS for thyroid-hypothyroid and fertility diagnosis datasets respectively in contrast with IFRFSMI. Moreover, for breast cancer, FSFrM and FRFS provide the similar size, whilst, for fertility diagnosis dataset FRFS produce similar size of the data when compared to the results presented by proposed method. From the recorded reduct in Table 1, it can be observed that our proposed technique is generating more reduced dimensions for most of the cases related to all the ten datasets rather than recently established powerful methods. We have presented the visualization of reduction process based on different methods in Fig. 1, which clearly indicates that our proposed method produces high percentage of overall feature elimination with the increment of total conditional features. Then, IBK and RARF are chosen to show the learning performances in terms of standard deviation with overall accuracies for the reduced datasets generated by four existing and our proposed techniques, where 10-fold cross validation is employed to avoid the overfitting. These results are reported in Table 2, where the ranks are outlined in the superscript of all the individual results. From the results available in Table 2, it is obvious that our proposed method is dispensing the better results in contrast with the results of other previous approaches regardless of reduced data produced by previous approaches, except the outcome for breast cancer and heart disease datasets. For breast cancer dataset, TIFRFS presents better outcome when compared to IFRFSMI by using both IBK and RARF, while, for heart disease dataset TIFRFS gave the best result with RARF. For colon and heart disease datasets, GIFRFS and TIFRFS leads to identical results as compared to IFRFSMI based results by IBK. Similar results are presented by RARF for fertility diagnosis and wdbc datasets based on the reduced datasets produced by FSFrMI and GIFRFS respectively in contrast with proposed method based reduced datasets. Entire results can be visualized by Figs. 2 and 3. These figures depict that proposed concept are very much effective for both low and high-dimensional datasets as the reduced datasets produced by this method always leads to increment of overall accuracies of the different learning algorithms regardless of their dimensionality size.

Our assumptions to verify the significance of our proposed method are as follows:

**Null Hypothesis:** All the employed methods are equivalent.

**Alternate Hypothesis:** There is significant difference among the employed methods.

Two widely accepted testing approaches namely Freidman test^88^ and Bonferoni Dunn test^89^ are applied to validate the significance of the presented method. Freidman test is used to perform comparative study of multiple models. Further, Bonferoni Dunn is employed to obtain which method is significantly different from proposed technique. The null hypothesis can be rejected at $\alpha \%$ level of significance if the values between their average ranks is higher rather than critical distance value. In the current study, average ranks by both IBK and RARF based on our proposed method are recorded as the minimum value (Table II). These values are clearly depicting the superiority of our established models. Moreover, F-statistics computed values based on IFRFSMI are obtained larger for both IBK and RARF when compared to F-tabular value. F-statistics computed values for IBK and RARF are 23.09 and 32.38 (Table II), whilst F-tabular value is 2.634 (F(4,36) = 2.634 at 5% level of significance). Therefore, based on Dunn Test our proposed method is found as significantly different.

**Table 1:** Dataset characteristics and reduct size.

Dataset	Instances	Features	Reduct size				
			FSFrMI	GIFRFS	TIFRFS	FRFS	IFRFSMI
Bank marketing	4521	16	10	12	15	15	14
Breast cancer	699	9	8	9	9	8	8
Dbworld-bodies	64	4702	97	128	187	88	8
Arcene	200	10000	453	287	303	268	169
Colon	62	32	24	27	21	18	8
Gsar-biodegradation	1055	41	31	36	29	33	25
Fertility diagnosis	100	9	8	6	8	7	7
Thyroid- hypothyroid	3163	25	11	17	19	15	12
Heart disease	294	13	11	10	10	12	9
Wdbc	569	21	17	14	18	10	8

**Table 2:** Comparison of overall accuracies with standard deviation for the datasets produced by FSFrMI GIFRFS, TIFRFS, FRFS, and IFRFSMI by using 10-fold cross validation.

Dataset	Classifier	FSFrMI	GIFRFS	TIFRFS	FRFS	IFRFSMI
Bank	IBK	84.75$\pm 2.88^2$	83.79$\pm 3.22^3$	83.01$\pm 2.19^4$	81.21$\pm 2.22^5$	86.28$\pm 1.29^1$
Marketing	RARF	87.23$\pm 3.11^3$	86.18$\pm 2.33^4$	87.59$\pm 1.21^2$	83.18$\pm 1.99^5$	89.37$\pm 0.86^1$
Breast	IBK	81.11$\pm 0.76^5$	86.24$\pm 3.83^3$	96.11$\pm 2.11^1$	84.29$\pm 2.89^4$	95.67$\pm 2.43^2$
Cancer	RARF	89.34$\pm 4.12^4$	93.34$\pm 3.02^3$	97.12$\pm 1.95^1$	88.66$\pm 3.22^5$	96.04$\pm 2.36^2$
Dbworld	IBK	89.16$\pm 7.27^4$	90.86$\pm 9.25^3$	91.89$\pm 7.23^2$	88.89$\pm 8.23^5$	94.74$\pm 8.28^1$
Bodies	RARF	90.25$\pm 6.88^5$	92.19$\pm 7.23^3$	93.55$\pm 7.89^2$	90.55$\pm 7.69^4$	97.21$\pm 6.00^1$
Arcene	IBK	71.47$\pm 10.25^3$	70.72$\pm 9.01^4$	72.09$\pm 10.12^2$	71.09$\pm 10.44^4$	74.00$\pm 9.53^1$
	RARF	75.69$\pm 7.55^3$	74.69$\pm 8.65^4$	77.55$\pm 9.28^2$	72.35$\pm 9.68^5$	83.45$\pm 9.09^1$
Colon	IBK	75.88$\pm 6.18^4$	78.12$\pm 5.84^{2.5}$	79.06$\pm 5.19^1$	73.06$\pm 7.88^5$	78.12$\pm 5.84^{2.5}$
	RARF	79.21$\pm 3.29^4$	80.41$\pm 2.99^3$	81.17$\pm 3.33^2$	77.17$\pm 3.33^5$	82.81$\pm 12.55^1$
Qsarbio-degradation	IBK	78.27$\pm 4.33^4$	77.69$\pm 3.87^3$	79.51$\pm 5.11^2$	75.87$\pm 4.45^5$	82.09$\pm 12.55^1$
	RARF	80.28$\pm 5.19^4$	81.33$\pm 4.66^3$	82.06$\pm 3.77^2$	79.16$\pm 4.78^5$	86.74$\pm 3.04^1$
Fertility diagnosis	IBK	83.21$\pm 9.88^2$	81.41$\pm 10.18^4$	83.17$\pm 9.99^3$	80.17$\pm 9.87^5$	84.30$\pm 9.98^1$
Thyroid- hypothyroid	RARF	87.20$\pm 6.68^{1.5}$	83.69$\pm 7.65^4$	85.23$\pm 5.77^3$	82.87$\pm 6.45^5$	87.20$\pm 6.68^{1.5}$
	IBK	92.33$\pm 3.22^3$	91.23$\pm 2.66^4$	95.16$\pm 2.77^2$	88.33$\pm 2.34^5$	97.87$\pm 0.69^1$
	RARF	95.21$\pm 2.88^3$	93.41$\pm 1.18^4$	97.17$\pm 2.55^2$	92.17$\pm 1.87^5$	99.11$\pm 0.46^1$
Heart disease	IBK	79.26$\pm 1.03^3$	78.46$\pm 2.28^4$	81.16$\pm 1.99^{1.5}$	76.25$\pm 2.99^5$	81.16$\pm 1.99^{1.5}$
	RARF	81.27$\pm 1.79^3$	80.38$\pm 1.23^4$	83.69$\pm 1.18^1$	78.98$\pm 1.55^5$	82.74$\pm 1.50^2$
Wdbc	IBK	95.68$\pm 0.28^2$	93.46$\pm 1.28^4$	95.16$\pm 1.87^3$	89.33$\pm 2.65^5$	96.06$\pm 0.11^1$
	RARF	96.41$\pm 2.28^4$	97.73$\pm 2.99^{1.5}$	97.69$\pm 3.19^3$	91.26$\pm 3.59^5$	97.73$\pm 2.99^{1.5}$
Average	IBK	3.20	3.55	2.15	4.80	1.30
Rank	RARF	3.45	3.35	2.00	4.90	1.30
F statistics	IBK	23.09				
	RARF	32.38				

#### Case study: an application to discriminate PL+ and PL- molecules

One of the prime applications of machine learning based methods in cheminformatics is the reduction of enormous chemical space with respect to some property of interest. The reduced chemical space can then be validated using wet lab based experiments, thus making the fidelity of machine learning methods of outmost importance.

**Figure 4:**
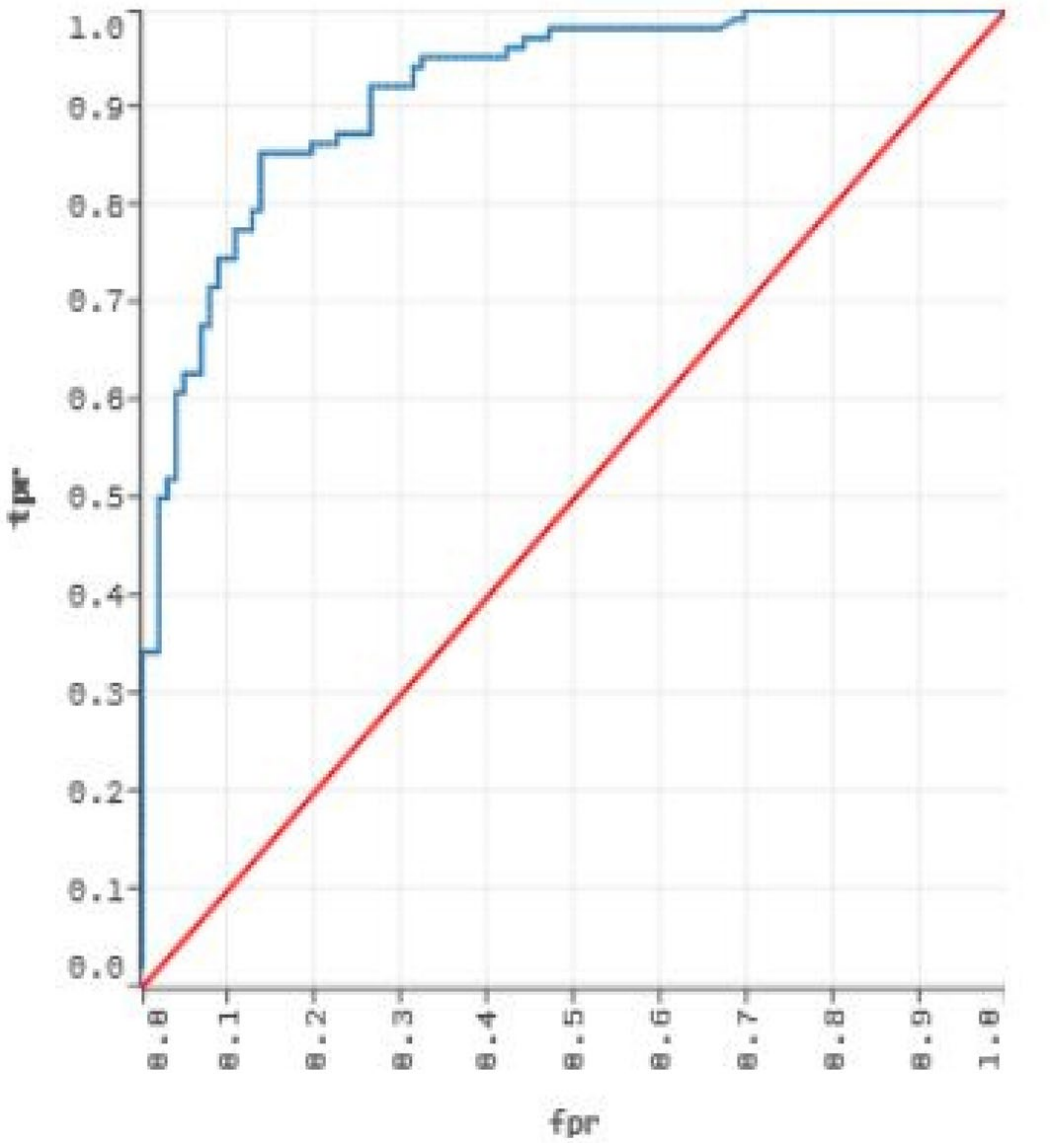
ROC for the RF algorithm on phospholipidosis dataset.

One of the hallmarks of phospholipidosis is the accumulation of phospholipids in the various types of tissues for eg. kidneys, eyes etc. mostly caused by cationic amphiphilic molecules. Highly accurate machine learning prediction models can facilitate in screening of phospholipidosis inducing compounds in early stages of drug discovery workflows, thereby reducing the cost and time associated with wet lab based experiments (Fig. 4).

The present methodology can open new possibilities for further research in early screening of phospholipidosis inducing molecules.

**Table 3:** Performance evaluation metrics of eight classifiers for original dataset consisting of PL+ and PL- molecules based on 10-fold cross validation.

Classifiers	Sensitivity	Specificity	Accuracy	AUC	MCC
Davie Bayes	75.5	81.4	78.4	0.828	0.570
SMO	81.4	85.3	83.3	0.833	0.667
IBK	82.4	80.4	81.4	0.806	0.628
RARF	81.4	85.3	83.3	0.908	0.667
PART	75.5	73.5	74.5	0.718	0.490
JRip	66.7	69.6	68.1	0.723	0.363
RandomForest	83.3	82.4	82.8	0.893	0.657
J48	74.5	74.5	74.5	0.769	0.510

**Table 4:** Performance evaluation metrics of eight classifiers for reduced dataset generated by proposed approach consisting of PL+ and PL- molecules based on 10-fold cross validation.

Classifiers	Sensitivity	Specificity	Accuracy	AUC	MCC
Navie Bayes	85.3	70.6	77.9	0.846	0.565
SMO	81.4	68.6	75.0	0.750	0.504
IBK	87.3	87.3	87.3	0.811	0.745
RARF	88.2	84.3	86.3	0.925	0.726
PART	71.6	72.5	72.1	0.778	0.441
JRip	74.5	80.4	77.5	0.811	0.550
RandomForest	84.3	84.3	84.3	0.915	0.686
J48	74.5	75.5	75.0	0.752	0.500

**Table 5:** Performance evaluation metrics of eight classifiers for original dataset consisting of PL+ and PL- molecules based on percentage split of 66:34.

Classifiers	Sensitivity	Specificity	Accuracy	AUC	MCC
Navie Bayes	70.3	84.4	76.8	0.831	0.548
SMO	70.3	87.5	78.3	0.789	0.581
IBK	75.7	84.4	79.7	0.789	0.599
RARF	78.4	81.3	79.7	0.893	0.595
PART	56.8	81.3	68.1	0.700	0.388
JRip	78.4	65.6	72.5	0.733	0.445
RandomForest	75.7	81.3	78.3	0.868	0.568
J48	70.3	71.9	71.0	0.735	0.420

**Table 6:** Performance evaluation metrics of eight classifiers for reduced dataset generated by proposed approach consisting of PL+ and PL- molecules based on percentage split of 66:34.

Classifiers	Sensitivity	Specificity	Accuracy	AUC	MCC
Navie Bayes	86.5	71.9	79.7	0.851	0.593
SMO	73.0	81.3	76.8	0.771	0.541
IBK	81.1	84.4	82.6	0.834	0.653
RARF	91.9	87.5	89.9	0.903	0.796
PART	78.4	84.4	81.2	0.890	0.626
LRip	54.1	93.8	72.5	0.735	0.512
RandomForest	81.1	87.5	84.1	0.904	0.684
J48	83.8	87.5	85.5	0.842	0.711

**Figure 5:**
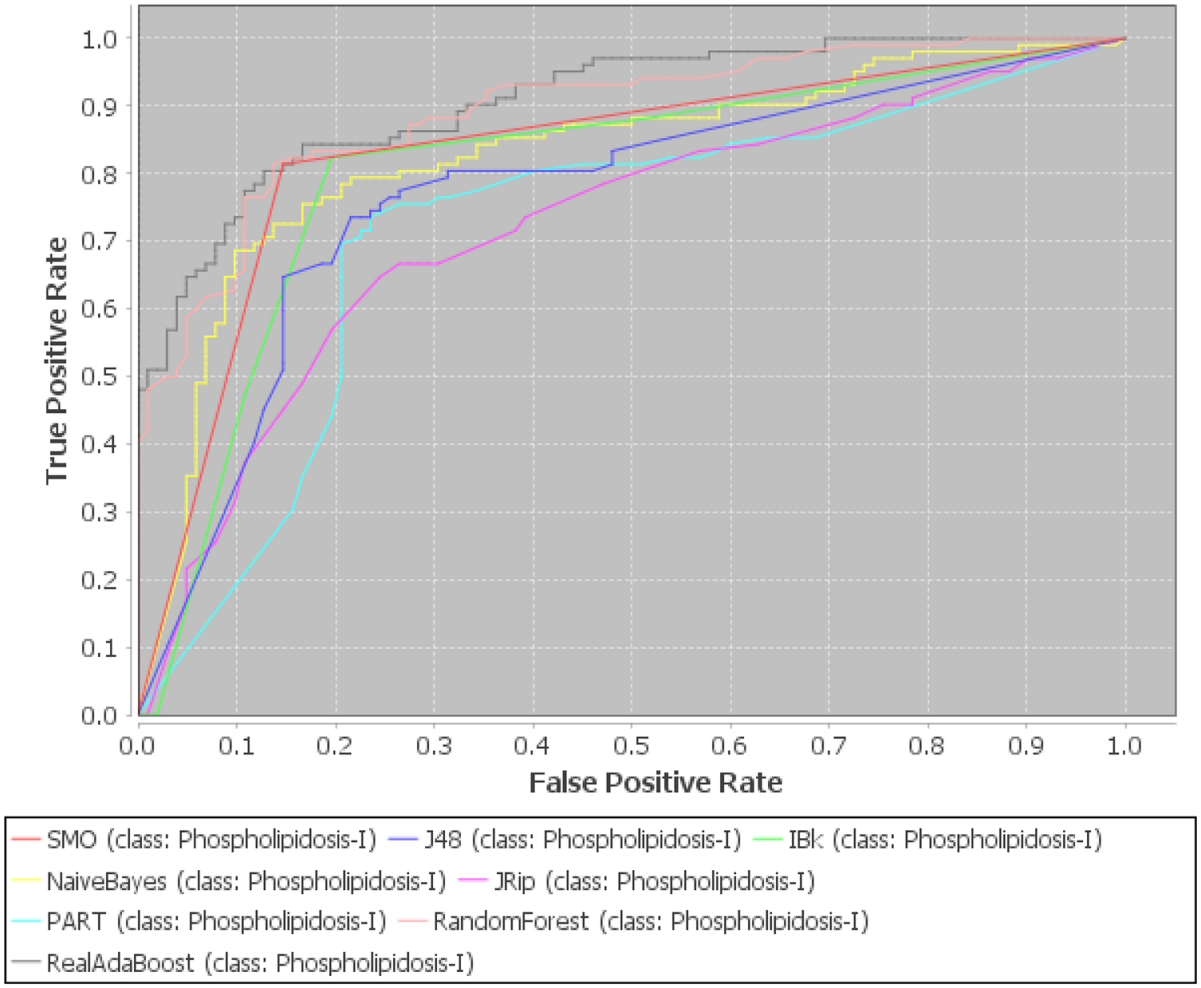
ROC curve for orginal dataset for various machine learing algorithms.

**Figure 6:**
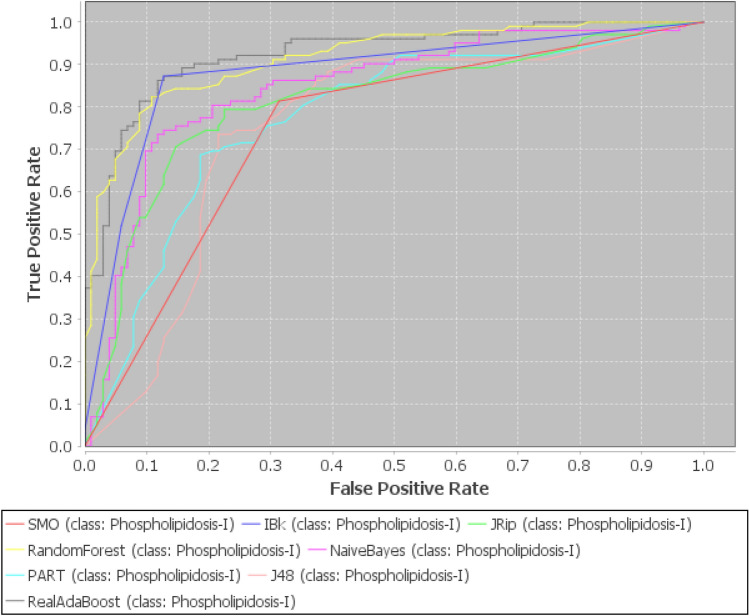
ROC curve for reduced dataset by various machine learing algorithms.

Now, our proposed approach is applied to Nath et al.^87^ dataset to produce the effective reduced form by minimizing noise, uncertainty, imprecision available in the data along with removal of redundant, and irrelevant attributes. Thereafter, seven classifiers from different categories are investigated to evaluate their performances over this reduced dataset based on sensitivity, AUC, Specificity, MCC, and accuracy, which have reported in Tables 3, 4, 5 and 6. Moreover, for original and reduced data, a commodious approach to represent theoverall performance measures of all the seven classifiers at the best decision threshold can be given by Receiver Operating Characteristic (ROC) curve, which furnishes a visual explanation of the classifiers performance. Figures 5 and 6 depict ROC curves for original and reduced dataset based on 10-fold cross validation. These figures indicate that RARF algorithm achieved the best AUC in comparison to all the other algorithms($>0.89$).

**Figure 7:**
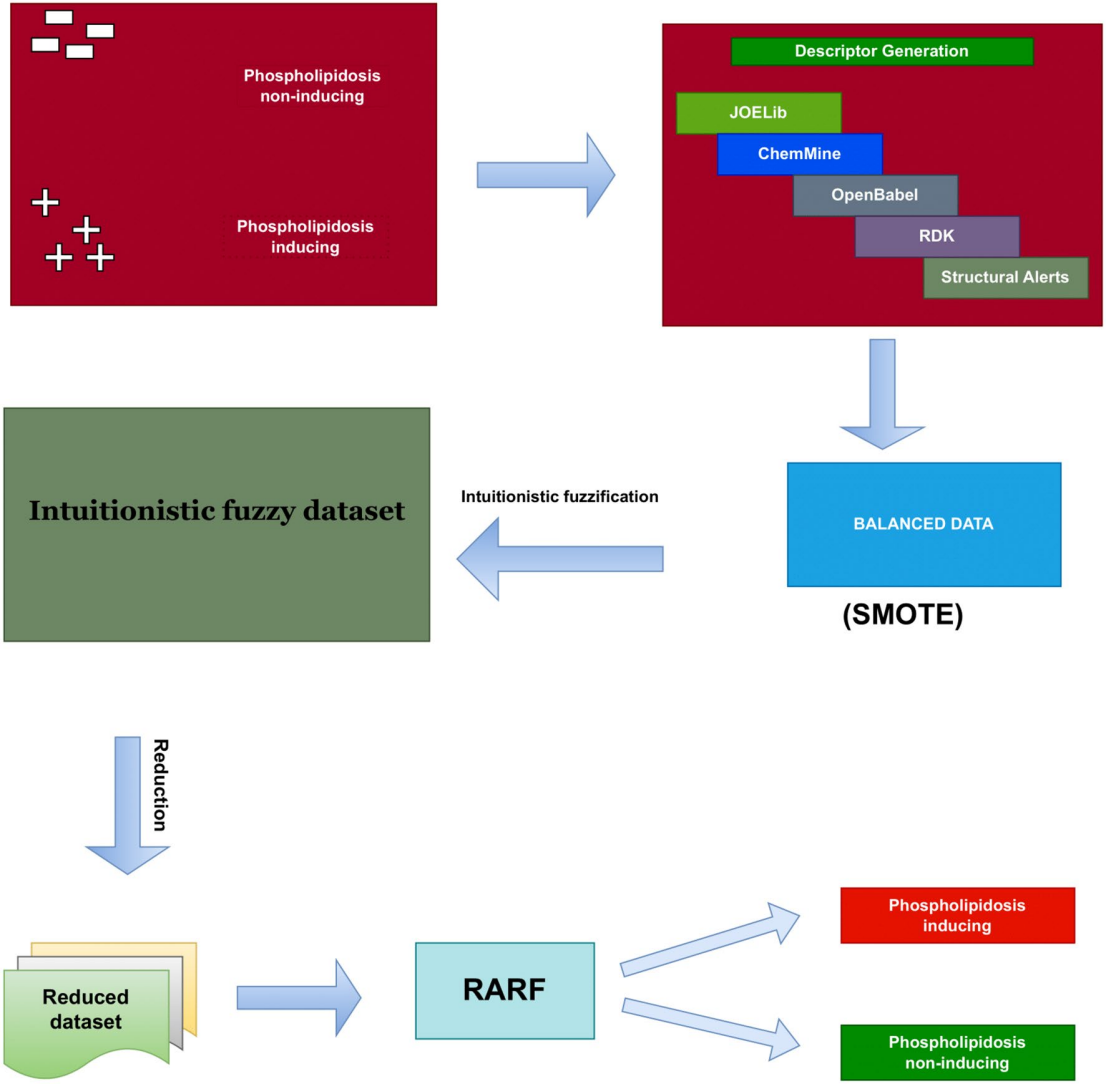
Schematic representation for generating classifier for phospholipidosis.

To compare with the performance evaluation metrics for the phospholipidosis dataset, we used the same package in R (https://https://cran.r-project.org/web/packages/h2o/index.html)as used in the original work (Nath et al.^87^). We used a grid search strategy to obtain the best hyperparameters for the random forest algorithm Hyperpaprametersntrees = c(20,50,100,500),max depth = c(20,40,60,80),sample rate = c(0.2,1,0.01). Further, we used the same of features (JOELib+Structural alerts), which are calculated using the ChemMine tools webserver (https://chemminetools.ucr.edu/). The dataset consisted of 102 phospholipidosis inducing compounds (positive samples) and 83 phospholipidosis non-inducing compounds (negative samples), thus constituting a total of 185 molecules. Schematic representation for entire process is given by Fig. 7. In the current methodology, we start the process with a dataset consisted of phospholipidosis positive molecules and phospholipidosis negative molecules. Then, descriptor generator converts the initial data into target data. Further, SMOTE is applied to obtain the balanced dataset. Next, this dataset is converted into intuitionistic fuzzy information system by using Tan et al.^57^ approach. Thereafter, our proposed feature subset selection method is applied to remove noise, vagueness, irrelevancy, redundancy, and uncertainty to obtain reduced dataset. Moreover, several classifiers are used to discriminate positive and negative classes. Finally, RARF is identified as the best performer.

**Table 7:** Perfomance evaluation metrics for the RF algorithm with previous method.

Classifiers	Sensitivity	Specificity	Accuracy	AUC	MCC
RF(h2o)	86.7	93.0	90.1	0.922	0.808
Nath et. al^87^	86.2	90.1	88.2	0.896	0.725

The performance evaluation metrics for the current method and the previous ensemble based method are presented in Table 7. The dataset preprocessing introduced in the current work resulted in enhanced performance evaluation metrics for the RF algorithm in comparison to the previously published results. Notably a 2 percent rise on overall accuracy is observed. As the dataset is slightly imbalanced, a rise in MCC for the current method proves the usefulness of the dataset preprocessing step. The ROC plot for the RF(h2o) model is presented in Fig. 4. An AUC value of 0.922 indicates an acceptable prediction model for phospholipidosis inducing molecules. In the end of the entire study, the list of abrreviations, signs, and symbols are presented in Table 8.

### Ethical approval

This article does not contain any studies with human participants or animals performed by any of the authors.

## Conclusion

Dimensionality reduction broadly aims to obtain a feature subset from existing original feature set by using certain powerful evaluation criterion. Since dimensionality reduction can produce efficient feature subset, where feature selection has found as an interesting central technique for data pre-processing in various beneficial and interesting data mining tasks. Conventional fuzzy rough set frequently incorporates dependency function as an evaluation criterion of feature subset selection. However, this method only maintained the maximum membership grade of a data point to one decision class and found to be unable in discarding later uncertainty and noise up to certain extent, which cannot characterize the classification error. To avoid these issues, we presented a novel intuitionistic fuzzy aided technique, where feature selection method is established by integrating information entropy with IF rough set concept.

- Initially, we established a hybrid IF similarity relation, which is further employed to present a novel IF rough joint and conditional entropies.
- Then, IF granular structure was introduced based on the proposed hybrid similarity relation.
- Thereafter, IF rough set model was described by using the aforesaid relation.
- Based on these entropies and granular structure, we suggested a mutual information idea to compute the significance of the feature subset for a decision class.
- Next, mathematical theorems are validated to justify the correctness of the proposed ideas.
- By using the significance notion a heuristic IF rough feature selection algorithm is represented. Then, we apply this heuristic algorithm on ten benchmark datasets to illustrate extensive experiments.
- Finally, proposed method is successfully employed to enhance the prediction performance for identifying PL+ and PL- molecules.

For dbworld-bodies dataset, our method has eliminated 99.83% features. Moreover, performance measures of learning algorithms were evaluated based on the reduced data produced by four existing and our proposed methods, where results clearly indicate superiority of the proposed technique. For thyroid- hypothyroid dataset, RARF has reported an accuracy of 99.11% and standard deviation of 0.46% for IFRFSMI based reduced dataset. For the discrimination of PL+ and PL- molecules, the best sensitivity is achieved based on 66:34 validation technique with 91.9%. The best overall result was obtained by RF(h2o) with sensitivity, specificity, accuracy, AUC, and MCC of 86.7%, 93.0%, 90.1%, 0.922, and 0.808 respectively.

The advantages of our proposed methodology can be outlined as bellow:

- This study presents a new hybrid similarity relation that can handle mixed data in intuitionistic fuzzy framework.
- Adaptive radius is computed in the recursive way from relation itself, which ensures the information loss.
- IF granular structure is implemented to deal with noise in mixed data as it is based on our proposed hybrid relation.
- IF rough mutual information is implemented to cope with noise and later uncertainty based on the proposed IF granular structure.
- This study presents a new methodology to discriminate PL+ and PL- molecules in an efficient and efficacious way.

In future, the proposed hybrid similarity relation can be improved by providing a more effective definition of adaptive radius. Further, inner and outer significance can be computed by assembling mutual information in robust IF rough framework to establish efficient approach to calculate the correlation between feature subset and class.

**Table 8:** The list of Abbreviations,Symbols, and Signs.

Abbreviations/symbols/signs	Explanation
IFS	Intuitionistic Fuzzy Set
FRS	Fuzzy rough set
DIFDT	Dominant intuitionistic fuzzy decision table
IFRS	Intuitionistic fuzzy rough set
IFMGRS	Intuitionistic fuzzy multigranulation rough set
MI	Mutual information
PL+	Phsopholipidosis positive
PL-	Phsopholipidosis negative
IFIS	Intuitionistic fuzzy information system
RARF	RealAdaBoost random forest
TRP	True positive
TRN	True negative
FLP	False positive
FLN	False negative
Sn	Sensitivity
Sp	Specificity
Ac	Accuracy
AUC	Area under curve
MCC	Mathews correlation coefficient
ROC	Receiver operating characteristic
SMO	Sequential minimal optimization
IBK	Instance based learner
FSFrMI	Feature selection based on fuzzy rough mutual information
GIFRFS	Granular structure based intuitionistic fuzzy rough feature selection
TIFRFS	Tolrence based fuzzy rough feature selection
FRFS	Fuzzy rough feature selection
IFRFSMI	Intuitionistic fuzzy rough feature selection based on mutual information
$\mu$	Membership grade
$\nu$	Non-membership grade
$\phi$	Hesitancy grade
$R_a^h$	Hybrid similarity relation
$\zeta _a$	Adaptive intuitionistic fuzzy radius
ET	Entropy
I	Mutual information
$\sum$	Summation
$\cup$	Union
$\cap$	Intersection
$\epsilon$	Epsilon
$\forall$	Forall
$\in$	Belong
$\Omega$	Significance

## Data Availability

The data supporting this study’s findings are available from the corresponding author (Mohd Asif Shah) upon reasonable request.
